# Unexplained isolated acute severe thrombocytopenia after surgery for a recurrent malignant retroperitoneal tumor presenting with colon perforation: A case study of a disastrous complication

**DOI:** 10.1016/j.ijscr.2020.01.046

**Published:** 2020-02-06

**Authors:** K.A. Boulas, A. Paraskeva, A. Triantafyllidis, A. Hatzigeorgiadis

**Affiliations:** Department of General Surgery, General Hospital of Drama, Drama, Greece

**Keywords:** Retroperitoneal tumor, Antiangiogenic therapy, Colon perforation, Surgery, Tumor growth, Thrombocytopenia

## Abstract

**Introduction:**

Tumor- or treatment- induced thrombocytopenia in solid cancer patients is common. In the postoperative setting, diagnosis of thrombocytopenia become more complex as infection, sepsis, drugs and transfusion come also into the equation.

**Presentation of case:**

Herein, the case an otherwise-healthy 71-year-old male patient with a sizable recurrent malignant retroperitoneal tumor under pazopanib admitted with colon perforation and submitted to emergency left colectomy with end transverse colostomy is presented. Immediate postoperative period characterized by massive primary tumor growth and isolated acute severe thrombocytopenia. The patient treated with combined prednisone, IVIg and platelets transfusion along with medication discontinuation with no response.

**Discussion:**

Sepsis-, drug- and heparin-induced thrombocytopenia, disseminated intravascular coagulopathy and secondary (sepsis-, drug-, transfusion- or tumor-induced) immune thrombocytopenia (ITP) were included in the differential diagnosis. Based on exclusion, secondary drug- or tumor-induced ITP was the most prominent diagnosis. Concomitant presentation of thrombocytopenia along with massive primary tumor growth made Kasabach-Merritt syndrome also a probable diagnosis. However, neither secondary ITP nor Kasabach-Merritt syndrome has previously been associated with a retroperitoneal tumor in the literature.

**Conclusion:**

Although management of thrombocytopenia depends on etiology, in our patient’s case the diagnosis of secondary ITP and directed management did not result in a successful outcome.

## Highlights

• Thrombocytopenia in cancer patients is correlated with poor prognosis.
• Drugs, sepsis, HIT, DIC, ITP are the most common causes of postoperative thrombocytopenia.
• Prompt diagnosis is essential as management varies considerably depending on etiology.
• Secondary ITP has not previously been associated with a retroperitoneal tumor.
• Kasabach-Merritt syndrome has not previously been associated with a retroperitoneal tumor.

## 1 Introduction

### 1.1 This work has been reported in line with the SCARE criteria [1]

Thrombocytopenia associated with solid cancer such as breast, lung, colorectal and ovarian cancer is common [2]. Tumor-induced thrombocytopenia in cancer patients may be the result of: (a) cytokines and transcription factors mutations and polymorphisms that are involved in platelet production; (b) malignant bone marrow infiltration; (c) paraneoplastic immune response. Treatment-induced thrombocytopenia in cancer patients may be the result of adjuvant chemotherapy and radiotherapy toxicity, administration of heparin and blood products transfusion [3]. When surgery is added, diagnosis of thrombocytopenia becomes more complex as infection, sepsis, drugs and transfusion come into the equation. Thrombocytopenia in cancer patients is correlated with poor prognosis; consequently, accurate and prompt diagnosis is essential as management varies considerably depending on etiology, severity and duration [4].

Herein, the case an otherwise-healthy 71-year-old male patient with a sizable recurrent malignant retroperitoneal tumor under antiangiogenic treatment admitted with colon perforation and submitted to emergency surgery is presented. The patient developed isolated acute severe thrombocytopenia in the immediate postoperative period; the most prominent diagnoses were: (a) sepsis- or drug- induced thrombocytopenia causing decreased bone marrow platelet production; and (b) secondary immune thrombocytopenia (ITP) (sepsis-, drug-, transfusion- or tumor-induced), heparin-induced thrombocytopenia (HIT) and disseminated intravascular coagulopathy (DIC) causing increased platelet destruction (Table 1). The present case report is educational as it describes the dynamic decision making process for differential diagnosis of postoperative thrombocytopenia as management varies considerably according to etiology, and unique due to the unusual presentation of secondary ITP associated with a retroperitoneal tumor.

**Table 1:** Common causes of postoperative thrombocytopenia.

Decreased platelet production	Increased platelet destruction	Platelet sequestration or dilution
Drugs	Sepsis	Significant intravenous fluid administration
Infection	Secondary (drug-, transfusion-, infection-induced) immune thrombocytopenia (ITP)	Massive red blood cell transfusion
Liver disease	Heparin-induced thrombocytopenia (HIT)	Splenomegaly
	Microangiopathy (thrombotic microangiopathy, disseminated intravascular coagulopathy)	
	Cardiopulmonary bypass	
	Continuous venovenous hemodialysis	

## 2 Presentation of case

**Fig. 1:**
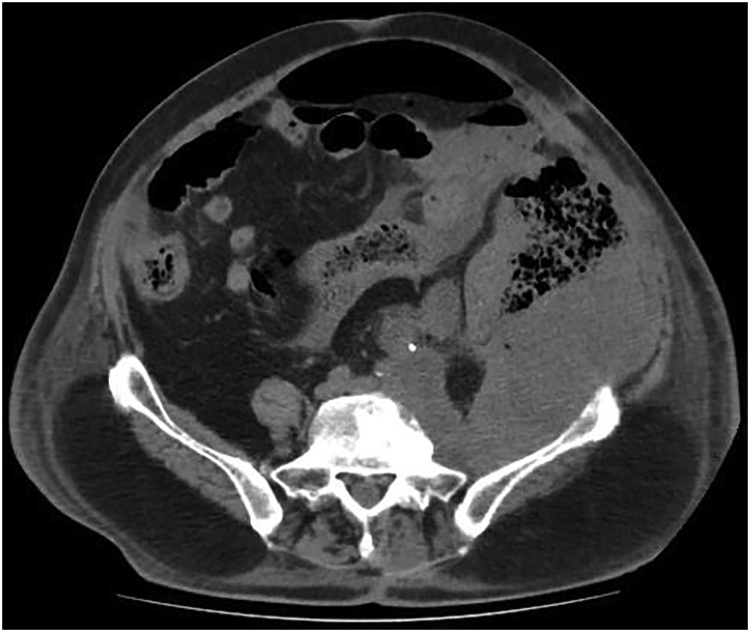
CT revealed the presence of a 16*10*12 cm solid left retroperitoneal mass with infiltration and perforation of the descending colon.

An otherwise-healthy 71-year-old male patient with a known sizable recurrent (the patient submitted elsewhere to a R2 resection to months prior) left retroperitoneal malignant fibrous histiocytoma under pazopanib (PO 800 mg daily) with infiltration of the left ureter under double J stent placed 3 months prior and the descending colon causing incomplete large bowel obstruction, admitted to the emergency department with symptoms and signs of peritonitis. Abdominal CT revealed the presence of: (a) a solid left retroperitoneal mass (approximate size16*10*12 cm) ; (b) infiltration of the left ureter with the presence of a double J stent; and (c) infiltration and perforation of the descending colon along with a large quantity of free intraperitoneal air and paracolic fluid (Fig. 1). Emergency laparotomy performed which revealed the presence of descending colon perforation and disseminated feculent peritonitis. The patient submitted to left hemicolectomy with end transverse colostomy and intraoperative saline peritoneal lavage. Postoperatively, pazopanib discontinued and tinzaparin (SC 4500 anti-Xa IU daily), omeprazole (IV 40 mg daily) and imipenem (IV 1gr q8h) were administered. Regarding surgical complications, postoperative period was uneventfull.

**Fig. 2:**
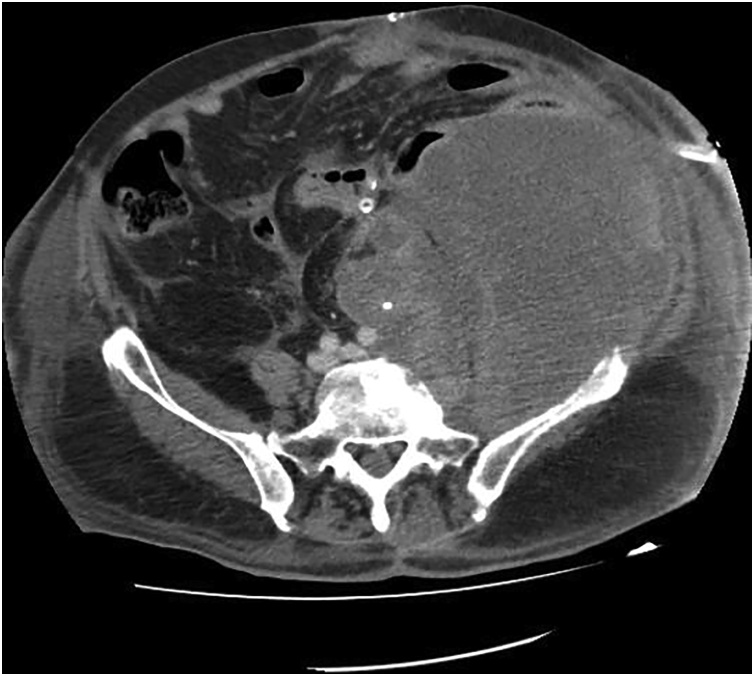
Postoperative CT showed rapid and massive primary tumor growth measuring approximately 20*14*15 cm in size with a 2.5-fold increase in volume.

On the 19th postoperative day, acute isolated severe thrombocytopenia (PLT < 10 × 10^9^/L) accompanied with hematuria occured. WBC count was normal, Hb count was 10.1 g/dL and regarding coagulation tests PT, aPTT, INR were normal, fibrinogen and D-dimmer was 198 mg/dL and 4.25 μg/mL respectively. Peripheral blood smear showed no red blood cells and leukocytes morphological abnormalities. The patient was afebrile; blood and urine cultures were negative. Chest and abdominal CT revealed (Fig. 2): (a) mild bilateral pleural effusion with bibasilar atelectasis; (b) no superficial or deep surgical site infection; (c) no thrombotic and hemorrhagic complications; and (d) progressive disease according to the RECIST 1.1 criteria with rapid and massive increase in the primary tumor volume (approximate size 20*14*15 cm with a 2.5-fold volume increase). Bone marrow biopsy revealed increased number and size of megakaryocytes. The patient treated with combined prednisone (IV 1 mg/kg), IVIg (400 mg/kg) and transfusion of one unit of apheresis platelets and two units of FFP daily along with discontinuation of tinzaparin and imipenem with no response over a 6-day period. The patient gradually died on the 27th postoperative day due to the onset of acute renal failure a couple of days earlier and respiratory failure.

## 3 Discussion

The present report describes a patient with a sizable recurrent malignant retroperitoneal tumor under antiangiogenic treatment admitted with colon perforation and submitted to emergency left colectomy with end transverse colostomy. In the immediate postoperative period, massive primary tumor growth and isolated acute severe thrombocytopenia observed. In this complex case, there were certain questions that needed to be clarified in the decision making process.

### 3.1 Question 1- what was the cause of bowel perforation?

Spontaneous bowel perforation is a potential complication in patients receiving antiangiogenic treatment. Qi WX et al. in a meta-analysis of 20 clinical trials including 5352 solid tumors patients receiving VEGFR inhibitors showed that the incidence of GI perforation was 1.3 % with a mortality of 28.6 %. Among cancer types, the highest incidence of bowel perforation was found in colorectal and ovarian cancer (0.9 % and 1 %, respectively). [5] Risk factors for antiangiogenic treatment-associated bowel perforation are presented in Table 2. Therefore, complete assessment of risk factors should be performed as the presence of active colitis, diverticulitis, peptic ulcer disease, extensive bowel infiltration and bowel obstruction are absolute contraindications for VEFG and VEFGR inhibitors use. [6]

**Table 2:** Risk factors related to bowel perforation in patients receiving antiangiogenic treatment.

Illnesses	Previous treatments
Bowel obstruction	Abdominal irradiation
Chemotherapy-induced colitis	Bowel surgery
Diverticulitis	NSAID
Peptic ulcer	Steroids
Tumor (intact primary tumor, tumor necrosis, transmural tumor)	Colonoscopy
Abdominal carcinomatosis	
Pancreatic primary cancer	
Ovarian primary cancer	
Rectosigmoid involvement	
Bowel involvement on CT	

Our patient had a known recurrent left retroperitoneal malignant fibrous histiocytoma firstly operated in 2017. Two months prior presentation, the patient submitted elsewhere to a second exploratory laparotomy. At that time preoperative CT and colonoscopy staging revealed the presence of descending colon infiltration with associated incomplete obstruction. The tumor considered unresectable; however no palliative loop transverse colostomy performed. Although the patient had risk factors for perforation such as intact primary tumor, bowel infiltration and obstruction, postoperative palliative targeted therapy with pazopanib initiated due to the limited therapeutic options. Conclusively, no adding a colostomy during the second operation, colon infiltration and obstruction in combination with VEGFR inhibitor treatment all contributed to colon perforation.

### 3.2 Question 2- what was the cause of the rapid and massive primary tumor growth?

Even a minor surgical trauma can influence pathophysiological processes that might promote postoperative metastatic spread and tumor recurrence. These pathophysiological processes encompass: (a) local effects including tumor seeding and wound healing response that can promote tumor cell migration, proliferation, differentiation, extracellular matrix remodeling, angiogenesis and extravasation; (b) local and systemic immunosuppression which impairs antitumor immunity and contributes to tumor cell survival; (c) cancer cell release into circulation and tumor cell dissemination by surgical manipulation of the tumor [7].

The effects of antiangiogenic treatment on tumor growth are well documented. However less is known about the effects of antiangiogenic treatment discontinuation on tumor re-growth. Iacovelli et al. in their retrospective study of 63 metastatic renal cell carcinoma who discontinued antiangiogenic treatment showed that tumor re-growth was related to the reason of discontinuation; re-growth was higher in patients who discontinued treatment because of disease progression or toxicity and lower in patients who discontinued treatment because of a sustained response; interestingly this study showed that the higher the growth rate, the shorter the survival [8]. In our patient’s case, surgical trauma and VEGFR inhibitor discontinuation resulted in rapid and massive primary tumor growth with 2.5-fold volume increase on repeated CT (Fig. 2).

### 3.3 Question 3- what was the cause of thrombocytopenia?

In our patient’s case, the most prominent diagnoses of thrombocytopenia were sepsis- and drug-induced thrombocytopenia, HIT, DIC and secondary (sepsis-, drug-, transfusion- or tumor-induced) ITP. Drug- and sepsis-induced thrombocytopenia was excluded as: (a) no sepsis criteria were present; (b) blood and urine cultures were negative; (c) chest and abdominal CT showed no imaging signs of pulmonary infection, superficial and deep surgical site infection; and (d) bone marrow biopsy revealed increased number and size of megakaryocytes [9]. HIT was excluded as the 4 Ts score was 3 suggestive of low clinical probability for HIT and therefore HIT lab testing was not ordered [10]. DIC was excluded as: (a) peripheral blood smear showed no red blood cells; (b) DIC score was 4 non suggestive of overt DIC [11]. The diagnosis of newly diagnosed secondary ITP, most probably drug- or tumor-induced, was supported by exclusion of other causes of thrombocytopenia as: (a) complete blood cell count revealed isolated thrombocytopenia in the era of chronic disease anemia; (b) peripheral blood smear showed no red blood cells and leukocytes morphological abnormalities; and (c) bone marrow biopsy revealed increased number and size of megakaryocytes without other significant abnormalities. However, non response to initial treatment with corticosteroids and IVIg did not support the immune nature of ITP [12].

In our patient’s case, the massive primary tumor growth, as a result of major surgical trauma and antiangiogenic treatment discontinuation, and the development of thrombocytopenia can be associated with Kasabach-Merritt syndrome. The diagnosis of Kasabach-Merritt syndrome can be supported by the presence of: (a) the aggressive, rapidly enlarging and probably highly vascularized retroperitoneal tumor; and (b) thrombocytopenia and mild coagulopathy including mild decrease in fibrinogen and increase in D-dimmer [13]. We believe that the concomitant presentation of massive primary tumor growth along with profound thrombocytopenia and mild coagulopathy was suggestive of platelet trapping and fibrinogen consumption within the abnormal tumor vascular endothelial architecture. However, neither secondary ITP nor Kasabach-Merritt syndrome has previously been associated with a retroperitoneal tumor in the literature.

## 4 Conclusions

Thrombocytopenia in cancer patients is correlated with poor prognosis. In our patient’s case, secondary drug- or tumor-induced ITP was the most probable diagnosis. The concomitant presentation of thrombocytopenia along with massive primary tumor growth made Kasabach-Merritt syndrome also a probable diagnosis. However, neither secondary ITP nor Kasabach-Merritt syndrome has previously been associated with a retroperitoneal tumor. Although management of thrombocytopenia depends on etiology, in our patient’s case the diagnosis of secondary ITP and directed management did not result in a successful outcome.

## Funding

None.

## Ethical approval

Ethical approval has been exempted by our institution.

## Consent

“Written informed consent was obtained from the patient for publication of this case report and accompanying images. A copy of the written consent is available for review by the Editor-in-Chief of this journal on request”.

## Author contributions

Boulas K was responsible for the study concept and design. Boulas K, Paraskeva A and Triantafyllidis A equally contributed in writing the paper. Hatzigeorgiadis A had the final approval of the paper.

## Registration of research studies

No unique identifying number requested for this case report.

## Guarantor

Hatzigeorgiadis A.

## Provenance and peer review

Not commissioned, externally peer-reviewed

## Declaration of Competing Interest

None.
